# Breast cancer classification based on breast tissue structures using the Jigsaw puzzle task in self-supervised learning

**DOI:** 10.1007/s12194-024-00874-y

**Published:** 2025-01-06

**Authors:** Keisuke Sugawara, Eichi Takaya, Ryusei Inamori, Yuma Konaka, Jumpei Sato, Yuta Shiratori, Fumihito Hario, Tomoya Kobayashi, Takuya Ueda, Yoshikazu Okamoto

**Affiliations:** 1 Department of Diagnostic Radiology, Tohoku University Graduate School of Medicine, 2-1 Seiryo-machi, Aoba-ku, Sendai, Miyagi 980-8575 Japan; 2 Department of Diagnostic Imaging, Tohoku University Graduate School of Medicine, 2-1 Seiryo-machi, Aoba-ku, Sendai, Miyagi 980-8575 Japan; 3 AI Lab, Tohoku University Hospital, 1-1 Seiryo-machi, Aoba-ku, Sendai, Miyagi 980-8575 Japan; 4 Department of Radiological Imaging and Informatics, Tohoku University Graduate School of Medicine, 2-1 Seiryo-machi, Aoba-ku, Sendai, Miyagi 980-8575 Japan

**Keywords:** Breast cancer, Mammography, Breast tissue, Deep learning, Self-supervised learning, Jigsaw puzzle

## Abstract

Self-supervised learning (SSL) has gained attention in the medical field as a deep learning approach utilizing unlabeled data. The Jigsaw puzzle task in SSL enables models to learn both features of images and the positional relationships within images. In breast cancer diagnosis, radiologists evaluate not only lesion-specific features but also the surrounding breast structures. However, deep learning models that adopt a diagnostic approach similar to human radiologists are still limited. This study aims to evaluate the effectiveness of the Jigsaw puzzle task in characterizing breast tissue structures for breast cancer classification on mammographic images. Using the Chinese Mammography Database (CMMD), we compared four pre-training pipelines: (1) *IN-Jig*, pre-trained with both the ImageNet classification task and the Jigsaw puzzle task, (2) *Scratch-Jig*, pre-trained only with the Jigsaw puzzle task, (3) *IN*, pre-trained only with the ImageNet classification task, and (4) *Scratch*, that is trained from random initialization without any pre-training tasks. All pipelines were fine-tuned using binary classification to distinguish between the presence or absence of breast cancer. Performance was evaluated based on the area under the receiver operating characteristic curve (AUC), sensitivity, and specificity. Additionally, detailed analysis was conducted for performance across different radiological findings, breast density, and regions of interest were visualized using gradient-weighted class activation mapping (Grad-CAM). The AUC for the four models were 0.925, 0.921, 0.918, 0.909, respectively. Our results suggest the Jigsaw puzzle task is an effective pre-training method for breast cancer classification, with the potential to enhance diagnostic accuracy with limited data.

## Introduction

Breast cancer is the most prevalent cancer among women worldwide in terms of the numbers of new cases and mortality rates [1]. Mammography has been widely used as a screening tool for early detection of breast cancer and has been shown to reduce breast cancer mortality [2, 3]. Despite its effectiveness, mammography-based screening faces limitations, such as a high rate of false positives and low sensitivity in patients with dense breast (DB) tissue [3].

In clinical practice, radiologists assess mammographic images by examining lesion-specific features as well as its relationship to surrounding breast tissue structures [4]. Lesion-specific features include subtle morphological changes at the tumor periphery and shapes of individual calcifications. Features related to surrounding breast tissue structures include the distribution of calcifications and architectural distortion of the breast tissue.

In recent years, deep convolutional neural networks (CNN) have been increasingly applied in the field of breast cancer screening using mammography [5–7]. In such CNN models, pre-training with classification tasks using large-scale natural image datasets, such as ImageNet [8], is commonly used to compensate for the scarcity of medical data [9]. However, traditional CNN models tend to rely heavily on local information, limiting their ability to utilize global information [10–13]. This disparity between the evaluation methods of radiologists and CNN models in breast cancer diagnosis underscores the necessity to analyze the association between lesions and surrounding breast structures.

Self-supervised learning (SSL) is a recent learning paradigm in deep neural networks that enables models to learn semantic features from unlabeled data [14]. The SSL pipeline is characterized by two tasks: the pretext task and the downstream task. The objective of the pretext task is to extract transferable representations useful for downstream tasks, such as classification, detection, and segmentation [15–17]. The Jigsaw puzzle pretext task, in which randomly placed patches are rearranged into their correct configuration, enables models to concurrently learn both features of images and the positional relationships within images [18]. It has been shown to be effective in medical imaging [17, 19–22]. Applying the Jigsaw puzzle pretext task approach in pre-training for breast cancer classification may help models to be close to that of clinical radiologists, which requires a comprehensive assessment considering both the characteristics of the lesion and of surrounding breast tissue structures.

The purpose of this study was to evaluate the effectiveness of the Jigsaw puzzle task in emphasizing breast tissue structures for breast cancer classification on mammographic images.

## Materials and methods

### Dataset

This study utilized the Chinese Mammography Database (CMMD) [23], which includes data from 1775 Chinese patients who underwent mammography examinations between July 2012 and January 2016, with biopsy-confirmed benign or malignant tumors. Of these patients, 826 had bilateral breast images and 949 had images of only one breast. The CMMD dataset contained both medial lateral oblique (MLO) and cranial caudal (CC) views of mammographic images, resulting in a total of 5202 mammographic images. In this study, only 2601 MLO view images, which have fewer blind areas on mammograms compared to CC view images, were analyzed. All images were acquired with digital mammography at a resolution of 2294 × 1914 pixels.

Based on image interpretation by a radiologist with over 20 years of experience in breast cancer imaging, the following cases were excluded from the 2601 images analyzed: those with phyllodes tumors (*N* = 6), neurofibromatosis (*N* = 2), lymphedema (*N* = 1), central venous ports inserted (*N* = 8), visible foreign bodies (*N* = 5), image artifacts that hindered diagnosis (*N* = 3), and those from breast cancer patients where lesions were unidentifiable (*N* = 140).

Ultimately, a total of 2436 images were included in our analysis, which were categorized as follows: 1167 images with breast cancer, 215 images with benign lesions, and 1054 breasts with normal breast tissue.

### Clinical interpretation of mammographic images

The CMMD dataset provides diagnostic labels indicating whether an image contains a benign or malignant lesion; however, it lacks detailed medical information within the images. To enhance the analysis from a medical perspective, all breasts were labeled with radiological findings (mass, calcification, distortion, normal), breast density (dense breast [DB], not dense breast [Not-DB]) and lesion masks based on the image interpretation of a radiologist with over 20 years of experience. For mass and calcification, the lesion mask that accurately captured the boundaries of the lesion were depicted. In contrast, for distortion, bounding boxes were employed to define the lesion mask, as these findings exhibit ambiguous boundaries with the surrounding normal breast tissue. When multiple findings were observed in a patient, images with each finding were counted independently.

**Fig. 1:**
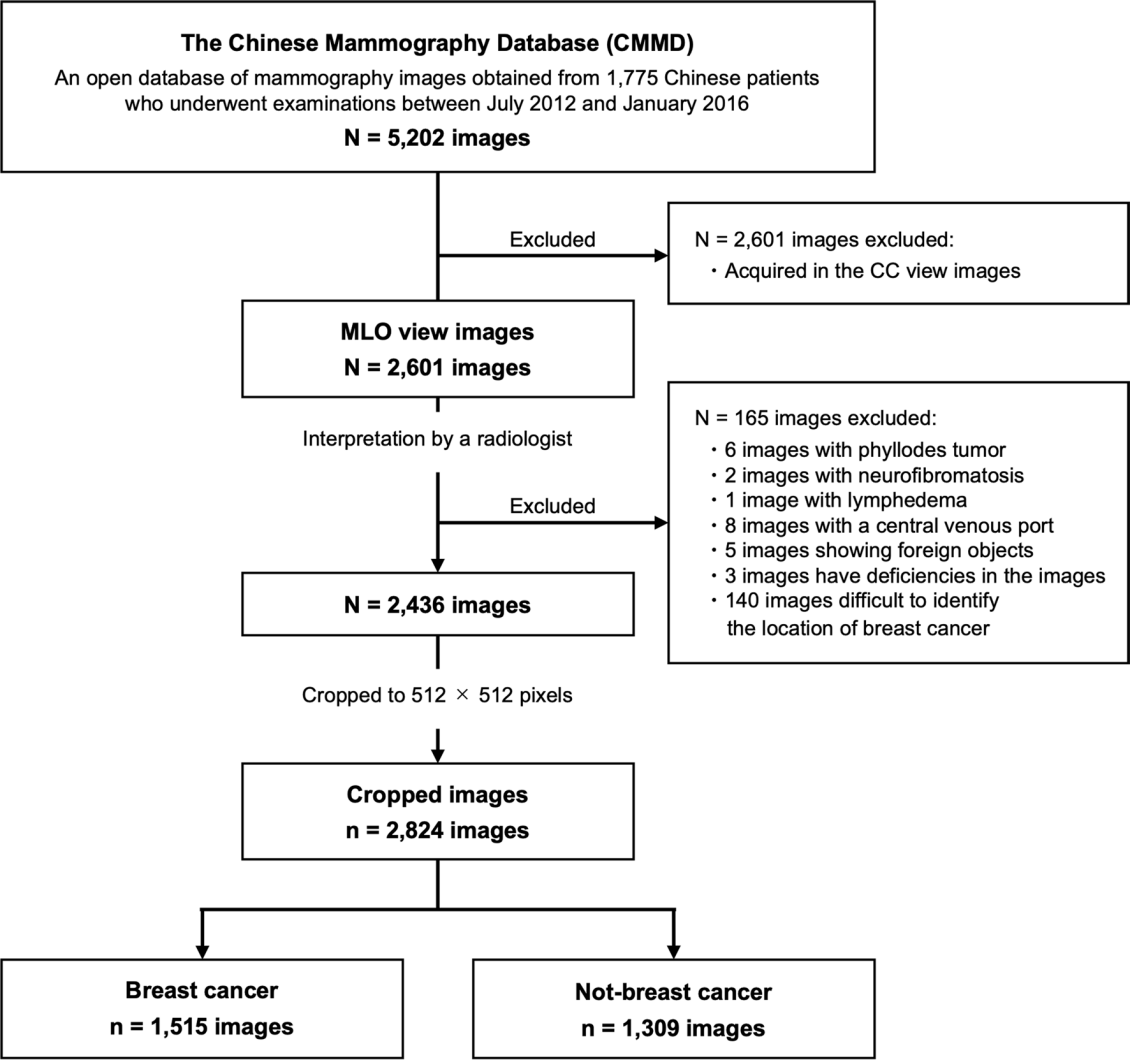
Flow chart of inclusion and exclusion process of mammographic database. Total 2824 mammographic images were analyzed in the present study

**Fig. 2:**
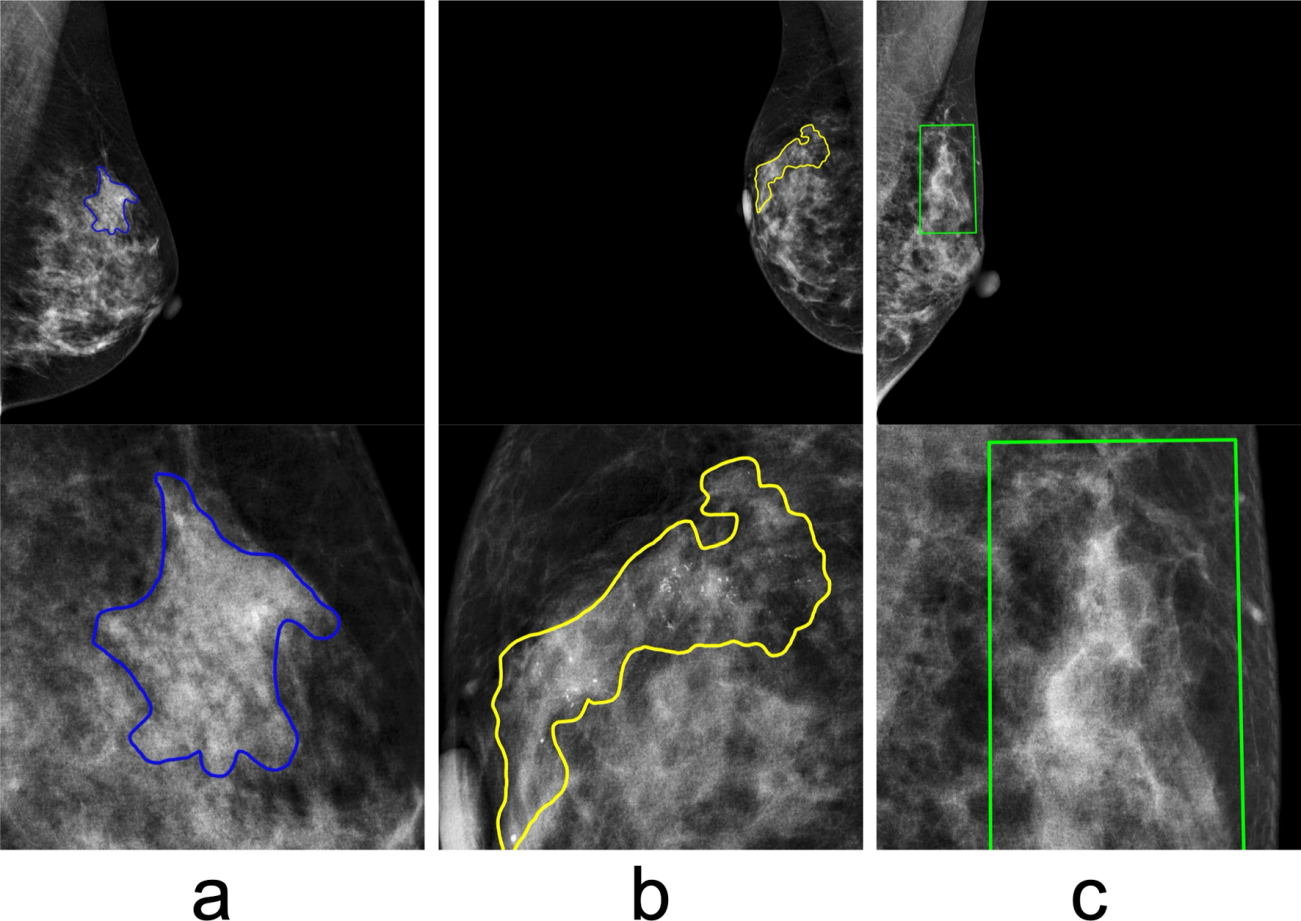
Examples of cropped images. The upper row and the lower row represents full-field images and cropped images, respectively. The radiological findings, breast density, and the percentage of the area occupied by the lesion masks in the 512 × 512 cropped images for each image are as follows: **a** (mass, DB, 20.4%), **b** (calcification, DB, 24.6%), and **c** (distortion, DB, 56.9%)

Using the lesion masks, original images were cropped to 512 × 512 pixels, centered on the lesion area. For breasts with normal breast tissue, the mammogram was binarized into breast area and background, and the outline of the breast area was extracted. Subsequently, to exclude the pectoral muscle, a 512 × 512 pixels image was randomly cropped from the bottom 80% of the breast area. Consequently, a total of 2824 images were used for our investigation, comprising 1515 images with breast cancer and 1309 images without breast cancer. Figure 1 illustrates the selection process, and Table 1 shows a breakdown of the datasets used in this investigation. Figure 2 shows examples of cropped images, where Fig. 2a–c represents mass, calcification, and distortion, respectively. The mean percentage of the area occupied by the lesion masks in the 512 × 512 cropped images were as follows: mass; 20.7%, calcification; 25.2%, and distortion; 57.5%.

**Table 1:** Breakdown of radiological findings and breast density information

Labels	Breast cancer	Not-breast cancer
Mass	954 images	186 images
Calcification	508 images	62 images
Distortion	53 images	7 images
Normal	–	1054 images
Dense breast (DB)	1236 images	1141 images
Not-dense breast (N-DB)	279 images	168 images

### The Jigsaw puzzle task in SSL

**Fig. 3:**
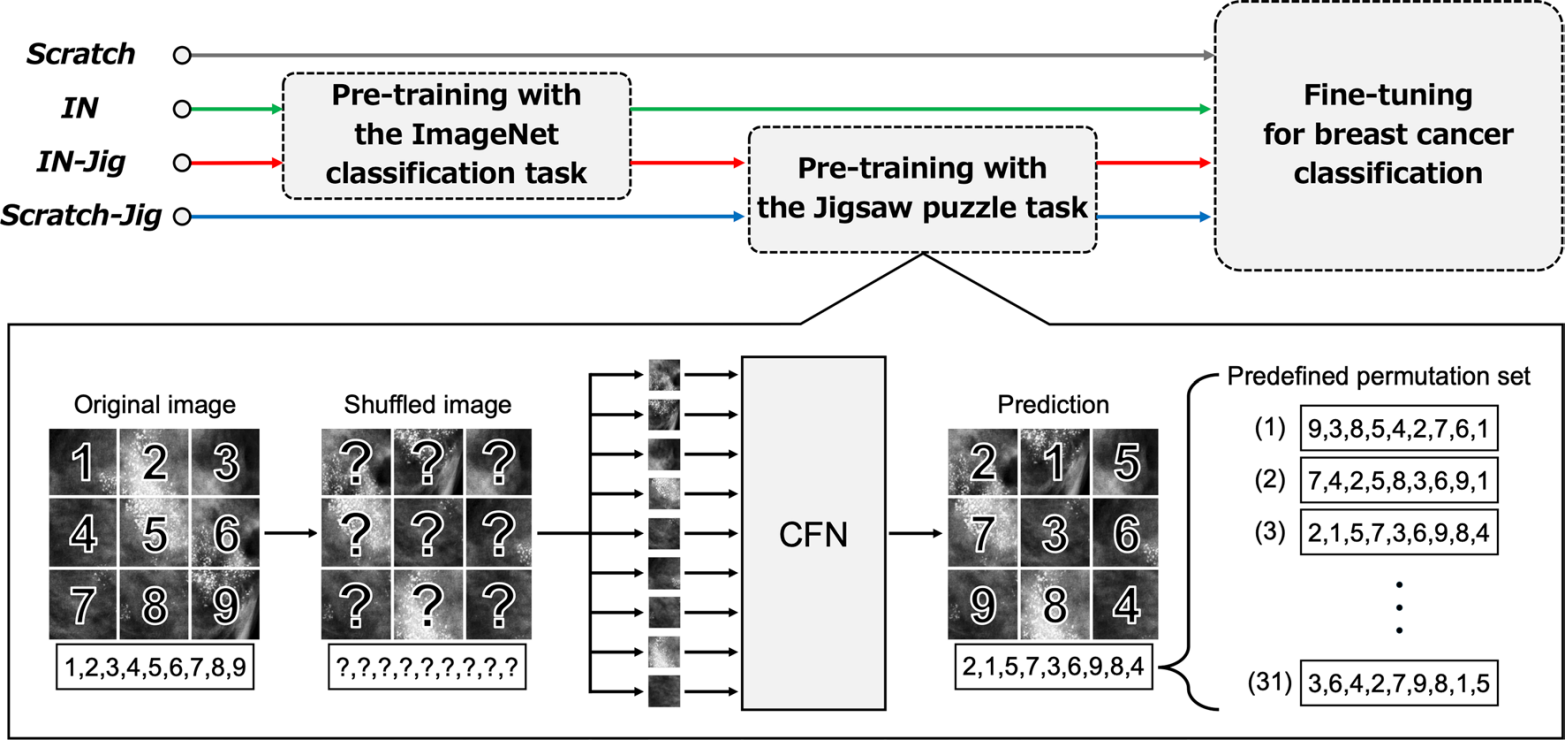
Pre-training pipelines and overall framework of the Jigsaw puzzle task in SSL. Abbreviations: *IN* = pre-trained only with ImageNet classification task; *IN-Jig* = pre-trained with both the ImageNet classification task and the Jigsaw puzzle task; *Scratch-Jig* = pre-trained only with Jigsaw puzzle task; *Scratch* = trained from random initialization without any pre-training tasks

The lower part of Fig. 3 shows the overall framework of the Jigsaw puzzle task in SSL used in this study. The input data for the Jigsaw puzzle task consisted of a set of patches cut from a mammographic image. Following the method described by Noroozi et al. [18], we left random gaps between the patches to prevent the model from solving the puzzle by simply matching low-level statistics (such as structural patterns and textures) at the edges of adjacent patches, ignoring the content within the patches themselves.

The set of patches was then reordered via a randomly chosen permutation from a predefined permutation set, and fed into the neural network. The neural network was trained to predict the specific permutation performed on the set of patch images. Although there are 9! (362,880) possible permutations with 9 patches for the 3 × 3 Jigsaw puzzle, a previous study has shown that it is not necessary to consider all 9! [18]. In the study of Vu YNT et al. [19], 31 different segment placement patterns were predefined, including the correct identity permutation and the top 30 permutations with the greatest Hamming distance from the identity.

The architecture used for the Jigsaw puzzle task was the context-free network (CFN) [18], which shares the same weights across all input patches. The features of each patch are extracted independently in each neural network and finally concatenated in a final fully connected layer.

### Comparison of pre-training pipelines

Figure 3 illustrates the pre-training pipelines compared in this study. To assess the effectiveness of the Jigsaw puzzle task and the effect of using the ImageNet pre-trained model, we compared the following four pipelines:

*IN-Jig* is the model pre-trained with both the ImageNet classification task and the Jigsaw puzzle task. The Jigsaw puzzle task was performed using the ImageNet pre-trained model. The neural network was then fine-tuned for binary classification to distinguish between the presence and absence of breast cancer.

*Scratch-Jig* is the model pre-trained only with the Jigsaw puzzle task, without utilizing an ImageNet pre-trained model.

*IN* is the baseline model pre-trained solely with the ImageNet classification task using random initial weights.

*Scratch* is the model that was trained for binary classification to distinguish between the presence and absence of breast cancer without any pre-training tasks.

### Implementation of the Jigsaw puzzle task

We adopted ResNet50 [24] as the architecture constituting the CFN. Each 512 × 512 pixels image was divided into a 3 × 3 grid consisting of patches of approximately 170 × 170 pixels. A 150 × 150 pixels-area was then randomly extracted from each patch and input into the CFN. We used the identical permutation and the top 30 permutations having the greatest Hamming distance from the correct permutation, following the reported method of Vu YNT et al. [19]. The mean Hamming distance of the total 31 permutations used in this study was 8.086. Adaptive moment estimation (Adam) was used for the optimizer and cross entropy was used as the loss function (learning rate = 0.001, weight decay = 0). The batch size was set to 128, and the model was trained over 100 epochs.

### Implementation of fine-tuning for breast cancer classification

For fine-tuning, binary classification was performed to distinguish between the presence and absence of breast cancer, using the same 512 × 512 pixels images as in the Jigsaw puzzle task. Adam was used for the optimizer and cross entropy was used as the loss function (learning rate = 0.001, weight decay = 0). The model was trained with a batch size of 64 over the course of 100 epochs.

### Evaluations

The overall performance of four models was assessed using receiver operating characteristic (ROC) curves, area under the ROC curve (AUC), sensitivity (true positive rate [TPR]), and specificity (true negative rate [TNR]). Furthermore, to elucidate the characteristics and trends of the model from a medical perspective, the models’ performance was also assessed for each group of radiological finding (mass, calcification, distortion, normal) and breast density (DB, Not-DB). Since AUC cannot be calculated for normal findings, the normal-true negative rate (normal-TNR) was used instead. Cutoff values for sensitivity, specificity, and normal-TNR were calculated using the Youden index (J), defined as J = maximum {sensitivity + specificity − 1}, which is considered to provide the best balance of sensitivity and specificity [25].

The accuracy of the Jigsaw puzzle task was assessed to compare the performance between the pretext and the downstream task. Gradient-weighted class activation mapping (Grad-CAM) [26] was implemented to visualize regions of mammographic images that were the most relevant for the network’s final classification. The Grad-CAM outputs were analyzed to assess how the regions of interest changed with use of the Jigsaw puzzle task.

To reduce bias due to a lack of data, fivefold cross-validation was employed. The dataset was divided into five equal parts, with four segments used for training and one for validation. This cross-validation process was repeated five times to calculate the mean validation accuracy. All data were separated by patient, ensuring a consistent process from pre-training to the downstream task. To mitigate the effect of randomness in fine-tuning, the fivefold cross-validation for the downstream task, breast cancer classification, was performed ten times. The mean performance scores and the mean ROC curves of ten trials were calculated. The statistical significance of the overall performance of AUC was assessed using a *t* test, with *IN* serving as the reference; a *p *value of less than 0.05 was considered statistically significant.

## Results

**Fig. 4:**
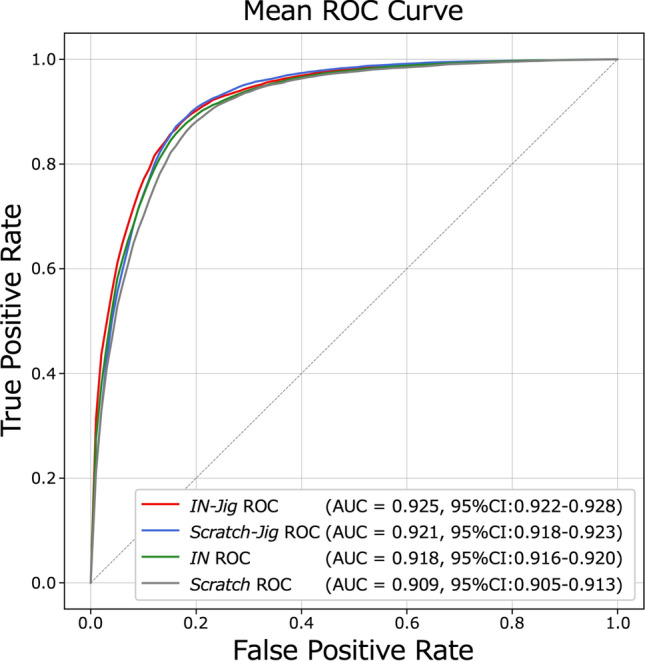
Mean ROC curves of four models

Table 2 presents the mean values of the AUC, sensitivity, and specificity, and Fig. 4 shows the mean ROC curves of the four models. The AUC of *IN-Jig* was 0.925, which was significantly higher than that of *IN* at 0.918 (*p* = 0.0048). The AUC of *Scratch-Jig* was 0.921, with no significant difference compared to the AUC of *IN* (*p* = 0.0516). The AUC of *Scratch* was 0.909, which was significantly lower than that of *IN* (*p* = 0.0011).

**Table 2:** AUC, sensitivity, and specificity of the four models

Model	AUC	Sensitivity	Specificity
*IN-Jig*	0.925 (0.922–0.928)*	0.879 (0.870–0.888)	**0.846 (0.838–0.855)**
*Scratch-Jig*	0.921 (0.918–0.923)	**0.890 (0.881–0.899)**	0.834 (0.828–0.841)
*IN*	0.918 (0.916–0.920)	0.882 (0.871–0.893)	0.831 (0.822–0.841)
*Scratch*	0.909 (0.905–0.913)*	0.879 (0.868–0.889)	0.817 (0.807–0.827)

The scores in the table are the mean of ten trials. Values in parentheses represent the 95% confidence interval

**Bold font** indicates the best score among the four models

The asterisk (*) indicates AUC that showed a significant difference compared to AUC of *IN*

**Fig. 5:**
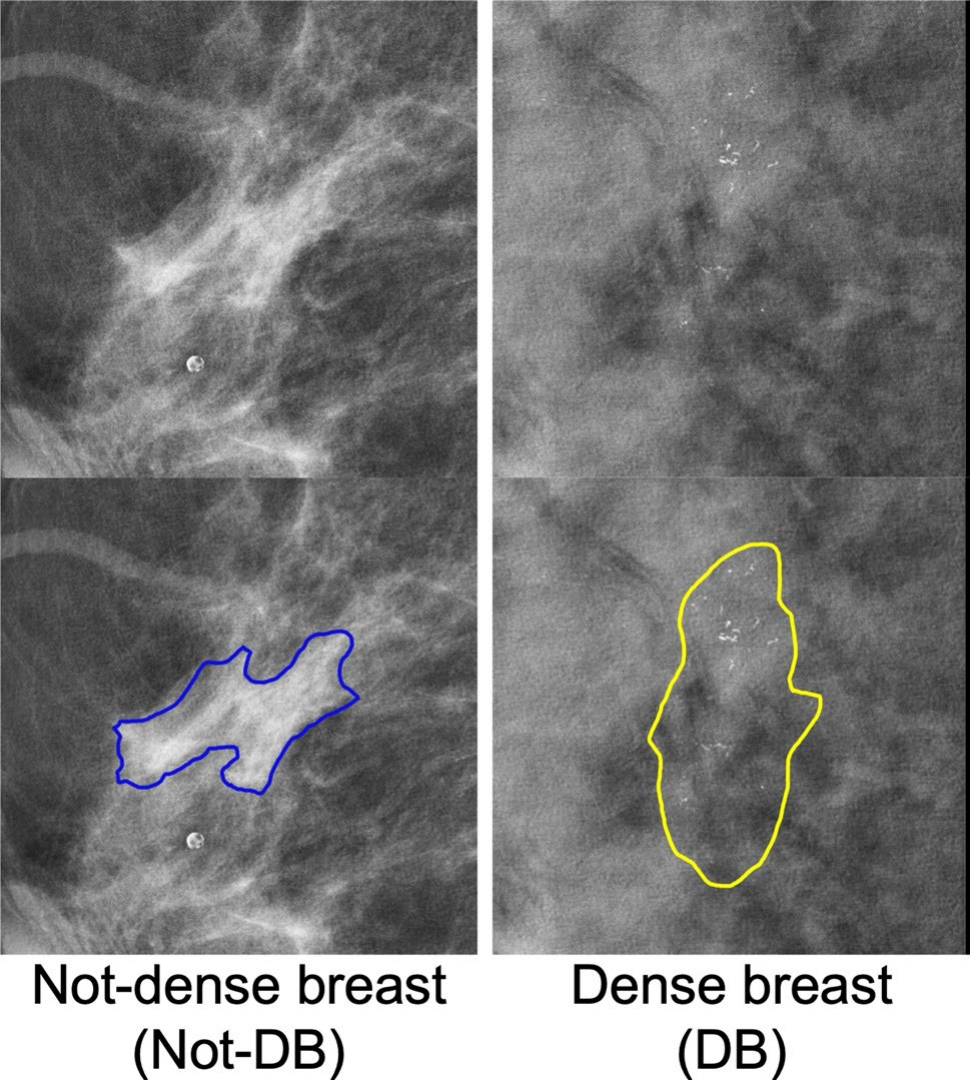
Samples of Not-DB and DB cases. The lower row represents images with the lesion masks added to the upper row. The images in the Not-DB and DB contain malignant tumors and malignant calcifications, respectively. In both images, *IN-Jig* correctly predicted, but *IN* incorrectly predicted

Tables 3 and 4 show the mean scores for each radiological finding and each breast density, respectively. In Table 3, mass-AUC and calcification-AUC of *IN-Jig* (0.766 and 0.783) were higher than those of *IN* (0.743 and 0.763), while *Scratch-Jig* (0.728 and 0.735) were lower than those of *IN*. In Table 4, DB-AUC was consistently lower than Not-DB-AUC for all models. *IN-Jig* and *Scratch-Jig* (0.916 and 0.913) had a higher DB-AUC than *IN* (0.909). Figure 5 illustrates samples of Not-DB and DB cases. In both cases, *IN-Jig* correctly predicted breast cancer, while *IN* incorrectly predicted those as not-breast cancer.

**Table 3:** Mean scores for each radiological finding

Model	Mass-AUC	Calcification-AUC	Distortion-AUC	Normal-TNR
*IN-Jig*	**0.766 (0.756–0.777)**	**0.783 (0.771–0.795)**	0.667 (0.618–0.716)	0.936 (0.934–0.943)
*Scratch-Jig*	0.728 (0.716–0.739)	0.735 (0.722–0.748)	**0.742 (0.697–0.787)**	**0.938 (0.929–0.942)**
*IN*	0.743 (0.737–0.748)	0.763 (0.748–0.777)	0.679 (0.635–0.722)	0.930 (0.923–0.936)
*Scratch*	0.697 (0.685–0.709)	0.755 (0.732–0.777)	0.674 (0.624–0.723)	0.926 (0.918–0.934)

The scores in the table are the mean of ten trials. Values in parentheses represent the 95% confidence interval

**Bold font** indicates the best score among the four models

**Table 4:** Mean scores for each breast density

Model	Not-DB-AUC	DB-AUC
*IN-Jig*	**0.963 (0.958–0.967)**	**0.916 (0.912–0.919)**
*Scratch-Jig*	0.951 (0.945–0.956)	0.913 (0.911–0.915)
*IN*	0.952 (0.948–0.955)	0.909 (0.906–0.911)
*Scratch*	0.947 (0.941–0.953)	0.900 (0.895–0.904)

The scores in the table are the mean of ten trials. Values in parentheses represent the 95% confidence interval

**Bold font** indicates the best score among the four models

Table 5 shows the accuracy of the Jigsaw puzzle task. *IN-Jig* (0.894) achieved a higher accuracy than *Scratch-Jig* (0.876).

**Table 5:** Accuracy of the Jigsaw puzzle task

Model	Accuracy of Jigsaw puzzle task
*IN-Jig*	**0.894**
*Scratch-Jig*	0.876

**Bold font** indicates the higher score between the two models

**Fig. 6:**
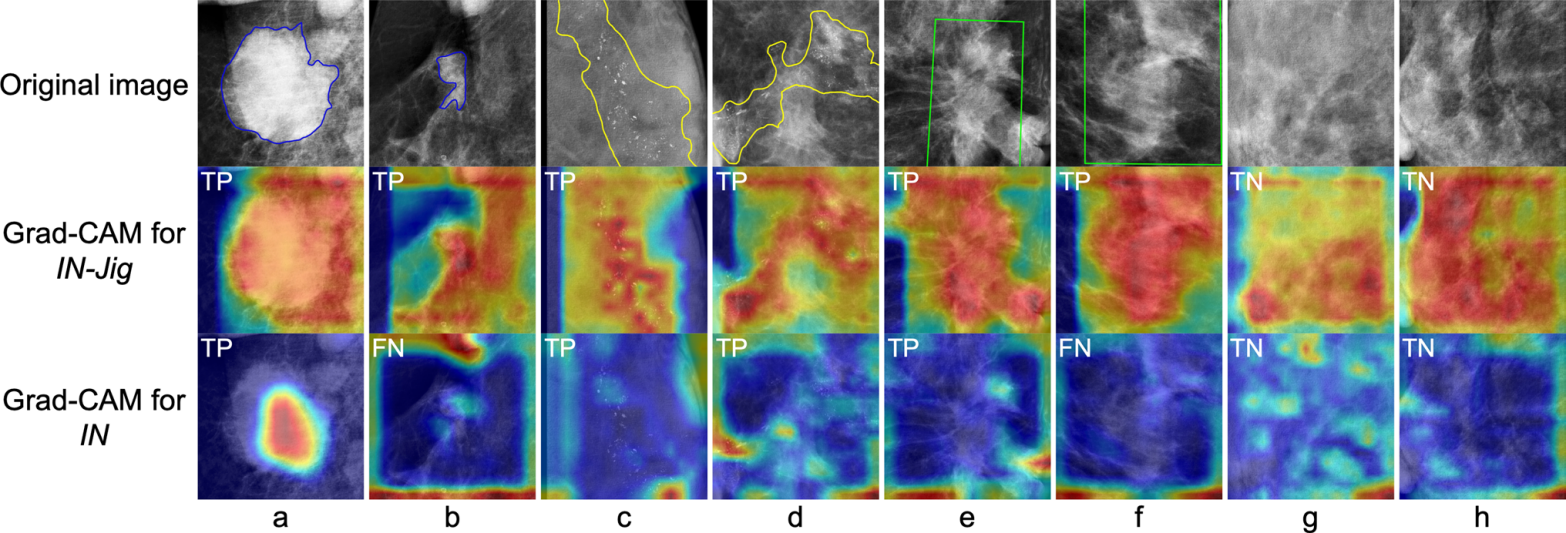
Visualized attention regions in the classification models with Grad-CAM. The upper row are originally-input images, and is described as follows: the blue regions in (**a**) and (**b**); malignant mass, the yellow regions in (**c**) and (**d**); malignant calcification, the green regions in (**e**) and (**f**); malignant distortion, and (**g**) and (**h**); normal breast tissue. The Grad-CAM highlighted areas are essential for classifying the image, with strongly emphasized regions marked in red and weakly emphasized areas in blue. The predicted results of *IN-Jig* and *IN* are shown in the top left of the Grad-CAM output image: true positive (TP), false negative (FN), and true negative (TN)

Figure 6 illustrates cases in which the application of the Jigsaw puzzle task led to a notable change in the regions of interest between *IN* and *IN-Jig*. *IN* made predictions by focusing on a part of the lesion or breast tissue. In the case of false negative (FN), *IN* focused on areas unrelated to the lesion. On the other hand, *IN-Jig* made predictions based on a wider area, including the entire lesion and surrounding breast tissue.

## Discussion

In the present study, *IN-Jig*, pre-trained with both the ImageNet classification task and the Jigsaw puzzle task, showed superior diagnostic performance in identifying breast cancers compared to *IN*, the conventional method. This suggests that the Jigsaw puzzle task, which emphasizes surrounding breast tissue structures, is effective for breast cancer classification on mammographic images. In addition, *Scratch-Jig*, pre-trained only with the Jigsaw puzzle task using approximately 2500 mammographic images, showed an AUC comparable to *IN*, which was pre-trained using approximately 1.3 million images [8]. These results suggest that pre-training with the Jigsaw puzzle task enables efficient learning of features useful for breast cancer classification, even with smaller datasets, and can further enhance diagnostic performance.

Although both *IN-Jig* and *Scratch-Jig* were pre-trained with the same Jigsaw puzzle task, there was a substantial numerical difference between *IN-Jig* and *Scratch-Jig* in mass-AUC and calcification-AUC. ImageNet pre-training likely enabled *IN-Jig* to capture more detailed features of mass and calcification during the Jigsaw puzzle task phase, while *Scratch-Jig*, pre-trained with the Jigsaw puzzle task using random weights as initial values, may not have fully utilized the detailed characteristics of the lesion. As a result, *Scratch-Jig* may have been biased toward diagnosis based on rough mammary gland structures, limiting its ability to improve mass-AUC and calcification-AUC.

Determining whether a breast is categorized as DB or Not-DB involves evaluating the entire breast, so this classification may not be directly applicable to cropped images such as Fig. 5. However, similar to the general challenge to distinguish between lesions and normal breast tissue in DB [27], DB-AUC of all models in this study was lower than Not-DB-AUC. In addition, DB-AUC of *IN-Jig* and *Scratch-Jig* were higher compared to *IN*. This indicates that the Jigsaw puzzle task is effective even for the more difficult task of classifying breast cancer in DB tissue.

*IN-Jig*’s higher accuracy on the Jigsaw puzzle task compared to *Scratch-Jig*, corresponded with *IN-Jig*’s superior AUC for breast cancer classification. Previous studies by Kornblith et al. [28] and Han et al. [29] have shown that higher pre-training accuracy often leads to better performance in downstream tasks. In this study, the same trend was observed, indicating that the Jigsaw puzzle task allows the model to learn generalized feature representations that are sufficiently beneficial for breast cancer classification.

The Grad-CAM results indicate that the Jigsaw puzzle task enabled the model to make predictions based not only on the lesion characteristics but also on surrounding breast tissue structures. These predictions are similar to the clinical diagnosis by radiologists, suggesting that breast cancer imaging diagnostic approach is able to be successfully incorporated into the model through Jigsaw puzzle pre-training.

This study has several limitations. First, the results were validated with the hyperparameters employed in prior studies, a single dataset, and a single neural network. Further investigation is needed to confirm the robustness and applicability of these results across different conditions. Second, we used cropped mammographic images of the lesion for the training and validation data. For large lesions, there was a possibility that the information of the surrounding breast tissue was not fully reflected due to the cropping of the image. Further investigation should explore models trained with whole breast images to evaluate their performance in a wider clinical framework.

## Conclusion

We investigated how the Jigsaw puzzle task affects breast cancer classification by analyzing in detail performance across different radiological findings, breast density, and regions of interest visualized with Grad-CAM. The results of this study suggest that using the Jigsaw puzzle task for pre-training improves CNN-based breast cancer classification on mammographic images. The combination of ImageNet and the Jigsaw puzzle task, *IN-Jig*, enhanced diagnostic accuracy by enabling the model to assess both the lesion and surrounding breast structures. Interestingly, *Scratch-Jig*, even without ImageNet, performed comparably to traditional methods, highlighting the potential of the Jigsaw puzzle task when working with limited data. Future studies are desired to validate these findings under various conditions to ensure broader applicability.

## Data Availability

The Chinese Mammography Database (CMMD) used in this study is available as open data via The Cancer Imaging Archive (TCIA) online data repository: 10.7937/tcia.eqde-4b16. The dataset to which a clinical interpretation was given in this study is available from the corresponding author upon reasonable request.

## Abbreviations

DB: Dense breast
Not-DB: Not dense breast
CNN: Convolutional neural networks
SSL: Self-supervised learning
CMMD: The Chinese Mammography Database
MLO: Medial lateral oblique
CC: Cranial caudal
CFN: Context-free network
Adam: Adaptive moment estimation
ROC: Receiver operating characteristic curves
AUC: Area under the ROC curve
TPR: True-positive rate
TNR: True-negative rate
normal-TNR: Normal-true negative rate
J: Youden index
Grad-CAM: Gradient-weighted class activation mapping
95% CI: 95% Confidence intervals
TP: True positive
FN: False negative
TN: True negative
*IN-Jig*: Pre-trained with both the ImageNet classification task and the Jigsaw puzzle task
*Scratch-Jig*: Pre-trained only with the Jigsaw puzzle task
*IN*: Pre-trained only with the ImageNet classification task
*Scratch*: Trained from random initialization without any pre-training tasks
